# Multiplex flows in citation networks

**DOI:** 10.1007/s41109-017-0035-2

**Published:** 2017-07-18

**Authors:** Benjamin Renoust, Vivek Claver, Jean-François Baffier

**Affiliations:** 1 National Institute of Informatics, Tokyo, Japan; 2 JST-ERATO Kawarabayashi Large Graph project, Tokyo, Japan; 3 Japanese-French Laboratory for Informatics CNRS UMI 3527, Tokyo, Japan; 4 University of California Berkeley, Berkeley, USA

**Keywords:** Citation network, Directed acyclic graph (DAG), Multiplex network, Metrics, Large network, Flow of knowledge

## Abstract

Knowledge is created and transmitted through generations, and innovation is often seen as a process generated from collective intelligence. There is rising interest in studying how innovation emerges from the blending of accumulated knowledge, and from which path an innovation mostly inherits. A citation network can be seen as a perfect example of one generative process leading to innovation. However, the impact and influence of scientific publication are always difficult to capture and measure. We offer a new take on investigating how the knowledge circulates and is transmitted, inspired by the notion of “stream of knowledge”. We propose to look at this question under the lens of flows in directed acyclic graphs (DAGs). In this framework inspired by the work of Strahler, we can also account for other well known measures of influence such as the *h*-index. We propose then to analyze flows of influence in a citation networks as an ascending flow. From this point on, we can take a finer look at the diffusion of knowledge through the lens of a multiplex network. In this network, each citation of a specific work constitutes one layer of interaction. Within our framework, we design three measures of multiplex flows in DAGs, namely the aggregated, sum and selective flow, to better understand how citations are influenced. We conduct our experiments with the arXiv HEP-Th dataset, and find insights through the visualization of these multiplex networks.

## Introduction

From the ancient times, knowledge has passed from individuals to others, each step leading to more discoveries and innovations. In modern times, the industrialization of research renders crucial to track this production of knowledge (Gibbons and Johnston 1974; Van Raan 2004). Indeed, it is important for the newly produced innovation to state on which ground it stands, enabling peers to judge the quality of the proposed innovation. An innovation must cite its influential sources to give credit to the work it was inspired from and to state its differences with the competing methods. This is one principle at the heart of the peer reviewing system enabling and validating the publication of new knowledge.

This process of citing sources is very important, because it makes explicit the transmission of knowledge from prior works to an innovation (Bornmann and Daniel 2008) — with the assumption that each new scientific publication is a container of innovation. Thankfully, this production of scientific knowledge can be easily captured in a citation graph. In this graph, nodes are publications citing other publications. This citation relationship is oriented and corresponds to a borrowing or derivation of knowledge. We suspect that the impact of a publication can be captured in this graph. The production of knowledge would then be represented as a growing process in a dynamic network.

Key for countries and organizations in modern science, the study of the production of knowledge is currently investigated from partial indicators that establish rankings and compare scientists. This gave rise to the development of many measures deriving from sociometrics (Waltman 2016) including age, field, and other cues. Three popular indicators are often used: the number of citations, the *h*-index (Hirsch 2005) — which originally measures both the productivity and the quality of an author —, and the impact factor (Reuters 2012) — which is a time-related average number of citations of a collection. These indicators are used for the evaluation of scientists, however they can be subject to controversy (Pendlebury 2009) and are designed to reflect only the productivity of a scientist rather than measuring the overall production of knowledge. One reason explaining these indicators’ popularity is their simplicity in terms of computation, as opposed to previous network analysis that was seen as too complex to deploy. However, progress in modern graph databases has grown, easing the analysis of dynamic networks (Cattuto et al. 2013).

Inspired by the seminal work from Strahler (1957) and from Hirsh (2005), we propose to bring a fresh look at the production of knowledge based on the analysis of flows in Directed Acyclic Graphs (DAGs). This view is not limited to the production of indicators but allows a more in-depth analysis of the process and diffusion of knowledge. The traditional indicators are very effective and it is important that our framework allows to establish them, while being easily extended. In addition, this DAG framework allows us to take a new perspective on citation relationships and introduces a multiplex network (Kivelä et al. 2014) model that emphasizes on co-citations (as opposed to the regular monoplex citation network).

In this work, we extend our proposition to join the different views on knowledge production in a recursive framework (Renoust et al. 2017) (which is covered in “Ascending flow in citation networks” section) to the analysis of multiplex citation networks and contribute with a new formalism, measures and experiments.

After discussing the literature in “Related works” section, the first part of this manuscript discusses the monoplex view of flows in citation networks (“Ascending flow in citation networks” section). In “Preliminaries” section, we introduce the Strahler numbers and the *h*-index in a generalized flow framework, and how those two notions belong to one greater notion of flow. We introduce our ascending flow in “Ascending flow in citation networks” section — modeled on the notion of flow of knowledge. We then discuss the parameters of this ascending flow and put it in relation with classical measures, while proposing a dynamic algorithm that allows for quick update. Finally, in “Experimental results” section we run experiments on a publicly available dataset, the arXiv HEP-Th (Gehrke et al. 2003). We then extend our ascending flow to the multiplex networks context from “The citation network as a multiplex network” section, which fits in our generalized flow framework. After defining our multiplex network in “Preliminaries” section, we propose three multiplex flow measures in “Multiplex extensions of the ascending flow” section, namely the aggregated, sum, and selective flows, with implementation in “Dynamic implementation” section. We present how we can visualize our networks in “Influence of layers in the multiplex citation network” section then repeat our experiments in “Experimental results” section on the same dataset and additionally lead in-depth examinations of publications before concluding in “Discussion and conclusion” section.

## Related works

### Monoplex approaches

The study of the production and transmission of knowledge has attracted quite a few scholars in the domains of social and economical science (Gibbons et al. 1994), with for example a focus on the population at the origin of production (Wuchty et al. 2007), and on transmission to business (Ernst and Kim 2002). These studies come *a posteriori* when observing controlled domains, with well known sociometric indicators. We are instead interested in the modeling of the production and diffusion of knowledge.

Many interesting attempts for modeling the production and diffusion of knowledge are actually focused on the *producers* of knowledge themselves, such as in multi-agent simulation (Cowan and Jonard 2001; Cointet and Roth 2007). In these models, the agents are interacting to produce knowledge, and the properties of the resulting interaction network of agents are the focus of analysis. The agents can then be tuned to produce different resulting networks, simulating real world policies (Mueller et al. 2015). The topology of the networks of people producing knowledge is often the main focus in related complex network research (Cowan R and Jonard 2004), with the goal to maximize diffusion in such networks (Alkemade and Castaldi 2005). In contrast, our focus is on the information produced itself and how it relates to previous works.

A good model for this is the citation graph. It mostly applies to academic research, but has found its way in complex network research. Numerous works actually focus on communities (Chen and Redner 2010), and on the characterization of the dynamics of the citation graphs (Gehrke et al. 2003). The closest to the spirit of our research would be the work by Hummon and Dereian (1989) who studied the main paths in the citation network in order to extract backbones and areas of interest. The question of the efficient implementation of these cues has been the focus of a previous contribution (Batagelj 2003). An extension of Hummon and Dereian’s original work has actually been applied to the study of the development of the *h*-index (Liu and Lu 2012). These methods are focused on the path produced by citations and use them as a base for bibliometrics, but without capturing the global flow of information. We propose in contrast a natural interpretation of flows in DAGs that can easily capture the same measures used for main path analysis.

Before introducing flows in DAGs, we need to mention one of the most cited work in scientometrics which is the *Hirsch index* (Hirsch 2005), globally known as the *h*-index. It originally applies to the authors and is designed to measures both the quantity and the quality of the authors’ production. Its definition is that an author with an *h*-index of *h* has published *h* articles which have been cited each at least *h* times. The version for publications is defined formally in “Preliminaries” section. It was rapidly followed by numerous variants and extensions (Waltman 2016). The most famous is possibly the *g*-index (Egghe 2006) that is the largest number such that the *g* articles with the most citations receive at least a total of *g* ^2^, averaging the importance of each article. Hirsh (2010) also proposes a more restrictive version called $\bar {h}$-index, normalized to domain or age. Other variants are designed with application-specific goals (Bucur et al. 2015). All-in-all, *h*-index based measures are measures to analyze the productivity of researchers, but do not allow for the in-depth analysis of production, in opposition to main path analysis approaches.

Our work roots its contribution in the analysis of flows in DAGs. Traditional max-flow approaches are quite far from what we define here, because nodes are always sources of information and edges have infinite capacities — we may be closer to multicommodity flows (Assad 1978). Instead, we mostly take our inspiration from a different notion of flows, in river streams, as defined by Strahler (1957). Limited to binary trees, this notion has seen a few extensions (Auber 2002; Delest et al. 2006; Herman et al. 1999) with applications to graph visualization. These versions use flows to highlight and extract most relevant paths in DAGs and trees, then relatively place elements one to another. We will use this approach and adapt it to the production of knowledge.

### Multiplex approaches

We later introduce the multiplex formulation of the citation network, which is a network made of multiple overlapping networks (each of which corresponds to a layer) connecting the same set of nodes (Berlingerio et al. 2013). Multiplex networks have been used to model citation networks mainly for clustering purpose (Boden et al. 2012; Dong et al. 2012; Renoust et al. 2014; Speidel et al. 2015). Boden et al. (2012) model the multiplex network from the keyword-publication associations in order to create clusters of publications of similar topic. Dong et al. (2012) share the same goal but mixes different layers from research domains, or different similarity links across the papers (diverse textual similarities, author similarity, and citations). Renoust et al. (2014) use an approach closer to Boden’s, with the different goal to find cohesive groups of co-authors in an author-publication network. Analysis of multiplex DAGs has also recently been proposed by Speidel et al. (2015) using the same arXiv HEP-Th dataset that we use, with the difference that layers are defined by year of publication. Beyond community detection, Pujari and Kanawati (2015) investigated the multiplex citation networks to predict the creation of link between authors: from the citation network they infer a multiplex network of authors and predict links, based on community structure. Our context differs in many ways from those works. The object we are studying, although from the same data, is very different. We keep the citation network as a support for modeling multiplex relationships, with the basic assumption of having a DAG structure. The network we construct presents a very large number of layers (one per co-citation) which is often an issue in the methods presented above. Our goals also differ, we are looking to find paths and remarkable nodes to evaluate the notion of publication impact.

The notion of flow has also been investigated in multiplex networks. Estrada et al. (2014) propose to study the dynamics of information in social networks through the communicability as a measure of flow to study the connectivity of layers. Although the goals and the object we study are quite different, this approach share commonalities with our sum and selective flows. Solé et al. (2015) proposes to study centrality and community structure from the point of view of information transfer within social networks while modeling the flow as a dynamically node-produced unit. Close to Solé et al.’s spirit, (De Domenico et al. 2015) have studied modular flows for random walkers within multiplex social networks derived from citations networks to identify communities of highly interacting researchers. Such approaches do not apply to our case: citations are only produced once and we do not study the dynamics between authors. Flows in their work are modeled to find centralities from random walks (which by definition can not be applied to DAG) with the goal to study community structures among authors.

Some digressing but interesting works focused on the study of time-related dynamics of flows and diffusion in multiplex networks are worth mentioning. Boccaletti et al. (2014) have produced a seminal work on the structural analysis of multiplex networks, however flows and diffusion are only approached from the dynamics side (close to the spirit of Gomez et al. (2013)). Pósfai et al. (2016) use maximum flows to study the controllability of 2-layer multiplex networks. Very recently Yu et al. (2017) proposed an analysis of the diffusion dynamics of binary states (such as the Prisoner’s Dilemma) in a multiplex social networks, investigating how different community layers influence this diffusion.

## Ascending flow in citation networks

We now introduce the notion of flow in a citation network inspired by the work of Strahler. We propose the ascending flow, parameterization, and experiments in the arXiv HEP-Th dataset.

### Preliminaries

We consider in our setting a citation graph *G*=(*V*,*E*) where *V* is a set of nodes — *publications* — and *E* a set of directed links, hereafter arcs, between nodes of *V*. An arc *a*(*p*,*q*)∈*E* represents a citation of publication *q* by publication *p*. We consider the graph as being directed acyclic (or DAG). Although real-world data may introduce a few cycles (arXiv edits leading to mutual citations), this is a marginal case that we will discard in our study.

In this setting, an author, journal, book, or proceedings can be modeled as collections of publications. Hence, by observing the collective impact of the collection we can characterize the influence of this set of publications. In other words, in our citation graph formalism — although we will not study them — collections are only sink nodes that can be sourced from the publications themselves. In this work, our measures of the impact of individual publications can be trivially reported to authors and collections.

#### **Definition 1**

For a publication *p*, its *in-neighborhood* $\mathcal {N}^- ({p})$ is the set of all the publications referring to *p*. The size of $\mathcal {N}^- ({p})$ is simply its in-degree *d* ^−^(*p*). The corollary implies that $\mathcal {N}^+({p})$, the *out-neighboorhood* of *p*, corresponds to all publications to which *p* is referring to (with a size *d* ^+^(*p*), its out-degree).

Strahler numbers measure the size of river streams depending on their flow of parent streams. If we recall the definition of Strahler’s numbers (Strahler 1957), in binary trees, it starts by assigning 1 to all leaf nodes, then iterates on parent nodes. If a parent node has at least two children with number *i*, then the node is assigned *i*+1, otherwise *i*. This number corresponds to the number of registers necessary to compute binary operations (Flajolet et al. 1979). The Extended Strahler (Auber 2002) does it for general trees (counting the register numbers of *n*-ary operations).

The *h*-index is defined as the maximum *n* such as *n* papers are cited *n* times. From this definition, the *h*-index applies in general trees of depth 3 and can actually be seen as a modified version of the Extended Strahler numbers. In this modification, a root node (e.g. an author) does not increase value from his maximum valuated nodes, but instead gets weighted by the maximum Extended Strahler number of his direct descendants (i.e. the publications).

Strahler numbers have been designed to define the size of river streams based on a hierarchy of dependent streams. Transmission of knowledge is very similar in that sense with publications being tributary to prior works they inherit from, and becoming in turn sources for later works — the *h*-index then captures the latter quantity. However, we want a finer measure which could capture the impact of a publication across all citations it has generated.

We defined above our citations graphs to be DAGs, and fortunately, Strahler numbers have also been extended to DAGs (Herman et al. 1999; Delest et al. 2006). Herman et al. (1999) proposes a generic framework to compute the importance *K* of nodes in DAGs — including Strahler numbers — such as:

1 $$ K(p) = \left\{\begin{array}{ll} \lambda,\text{if }\mathcal{N}^- ({p}) = \emptyset \\ F\left(K(a_{1}),\ldots,K(a_{d^{-}(p)})\right), \text{otherwise}, \end{array}\right.  $$

where *λ* designates a constant for terminal cases (leafs, often *λ*=1), $a_{i}\in \mathcal {N}^- ({p})$ represents the ancestors of node *p*, and *F* is an application depending on the values $K(a_{1}),\ldots,K(a_{d^{-}(p)})\phantom {\dot {i}\!}$. To simplify the notations, we denote $F(\mathcal {N}^- ({p}))=F(K(a_{1}),\ldots,K(a_{d^{-}(p)}))\phantom {\dot {i}\!}$.

This framework is nothing but a generic recursive framework, however it allows us to redefine other measures through it. In this context, counting the number of citations would only require to modify the application $F(\mathcal {N}^- ({p}))$, such as $F(\mathcal {N}^- ({p})) = |\mathcal {N}^- ({p})| = d^{-}(p)$. Similarly, the Strahler number of a node *p* is then defined as:

2 $$ F(\mathcal{N}^- ({p}))= \left\{\begin{array}{ll} 1,\text{if}~ d^{-}(p) = 0\\ \max\limits_{q\in\mathcal{N}^- ({p})}(K(q))+ \left\{\begin{array}{ll} d^{-}(p)-1~\text{if all values}\ K(q)\ \text{are equal} \\ d^{-}(p)-2~\text{otherwise} \end{array}\right. \end{array}\right.  $$

The application for the *h*-index then becomes:

3 $$ F(\mathcal{N}^- ({p}))= \left\{\begin{array}{ll} 0,\text{if}~ d^{-}(p) = 0\\ \max\limits_{X\subset \mathcal{N}^- ({p})}\min\limits_{q\in X}(d^{-}(q),|X|) \end{array}\right.  $$

Strahler numbers, number of citations, and *h*-index impose a discrete limit in depth which is conceptually an issue — there is no reason not to look for all the extended consequences of a publication. Instead, Herman et al. (1999) propose in their framework a *Flow metric* for DAGs to emphasize the distribution of information to their successor such as:

4 $$ F(\mathcal{N}^+({p}))= \left\{\begin{array}{ll} 1,\text{if}~ d^{+}(p) = 0\\ \sum\limits_{i} \frac{K(s_{i})}{d^{+}(s_{i})}\ \text{otherwise}, \end{array}\right.  $$

where $s_{i}\in \mathcal {N}^+({p})$ represent the successor of *p* (instead of the ancestor *a*_*i*_). Note that this defines a *descending* flow measure, which captures how much information all nodes in the network receive.

Before we provide our own measure of flow, two natural definitions help defining our framework and its integration with existing metrics.

#### **Definition 2**

*(Related)* Two articles *p* and *q* are said to be related if and only if there exist a path from *p* to *q* or from *q* to *p*. They are *k*-related if they are related and if the shortest path between them is at most of length *k*.

#### **Definition 3**

*(k-diffuse)* A measure of a node *p* is *k*-diffuse when it limits its computation to a subgraph composed of the *k*-related nodes of *p*.

Our base measures, the *h*-index and the number of citations, are then respectively 2- and 1-diffuse by definition.

We provide now a base measure called *ascending flow* and discuss its complexity. We then extend it to several variants, such as one that is restricted in depth, hence a better fit to dynamic computation.

### Ascending flow in citation networks

We can now model the stream of knowledge as a flow in our citation network. Indeed, each node — being a publication — produces some information and this production of information gives credit to their ancestors (in history, or successors in the DAG) as they refer to them. This translates into the framework as:

5 $$ F(\mathcal{N}^+({p}))=\sum\limits_{i} \frac{K(s_{i})}{d^{+}(s_{i})}+\lambda_{p}  $$

where *λ*_*p*_ represent the information created by the publication *p* — in practice we set *λ*_*p*_=1. Hence, the more a publication is influential the more credit it will propagate to its ancestors. In contrast to the previous *Flow metric*, our ascending flow is not only applied to the reversed DAG, but is also equivalent to the sum of the flows computed for each sub-DAG induced by each node.

The ascending flow, formalized above, can be implemented as Algorithm 1. It is important to note that each arc is visited only once and that the total number of visits of all nodes is also equal to the number of arcs. The time complexity of our algorithm is then *Θ*(*m*) where *m* is the number of arcs. This key property is inherent to the DAG nature of our citation network. Another straightforward property of DAGs is that $m \leqslant \frac {n(n-1)}{2}$ where *n* is the number of nodes. However, even a linear time complexity is often too costly for large dynamic networks.


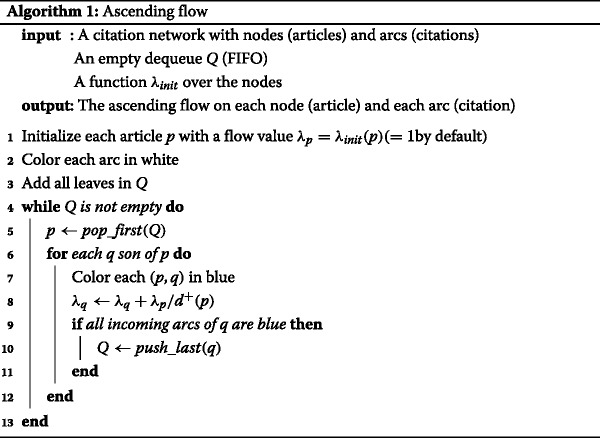


### Depth restriction and dynamic graph

**Fig. 1:**
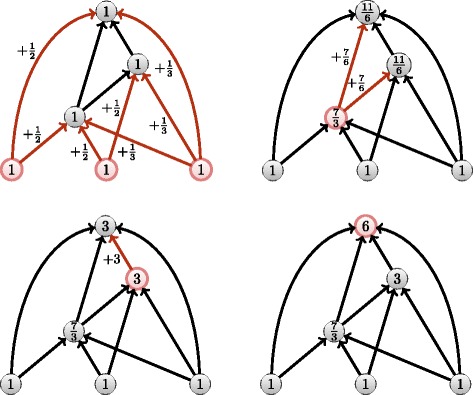
Ascending flow algorithm: step by step

As discussed above, one issue of computing the ascending flow of a node *p* from our definition is that it needs the computation of all its predecessors own influence. Such a constraint is expansive in the context of a dynamic network, for instance citation networks — in the case of citation network, publication are usually added, not removed. To adapt our previous algorithm, we first need to introduce an update function starting from a single leaf (a new publication). We consider the network initializes as in Algorithm 1 but for the flow value on the nodes — that is kept between the updates. We then propagate upwards the flow value in all the subgraphs defined by the ancestors of this publication (Fig. 1).

**Fig. 2:**
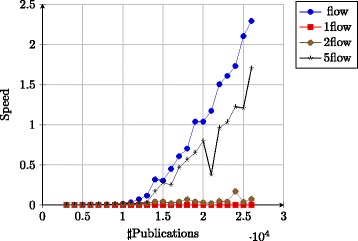
Speed comparisons of our algorithm in case of *k*-diffuse limitations

Recall the diffuse property in Definition 3, the ascending flow appears *∞*-diffuse. In the real-world, we can consider that a publication that came a few generations after an original will relatively diverge from the original one, and would marginally contribute to the influence of the previous publication. The *k*-diffusion property can then take two forms: either we choose a generational limit *k* that cuts the added influence of nodes generated *after* *k* generations, or we can set an *evanescence* coefficient that progressively attenuates the contribution of a publication over its ancestors. In the case of a dynamic citation network, a *k*-diffuse measure is very quick to compute when *k* is a small constant as displayed in Fig. 2.

This depth parameter additionally allows us to reconnect with known measures. The *h*-index is 2-diffuse and it would not make sense to extend its definition. In turn, the number of citations — which is also the in-degree (*d* ^−^(*p*)) — is 1−diffuse and can be easily translated in a *k*-diffuse measure, the *k*-degree, which would be the number of publications created until generation *k*. Then, an *∞*-degree would be the number of all publications seeded by *p* even indirectly.

### Experimental results

We now apply our framework on a real-world setting. We used an available citation graph from 2003 KDD Cup: arXiv HEP-Th^1^ (Gehrke et al. 2003). It consists in an archive of 27,770 publications with 352,807 (internal) citations from the well-known arXiv website of pre-prints in the domain of high energy physics theory, archived between January 1992 to April 2003.

**Fig. 3:**
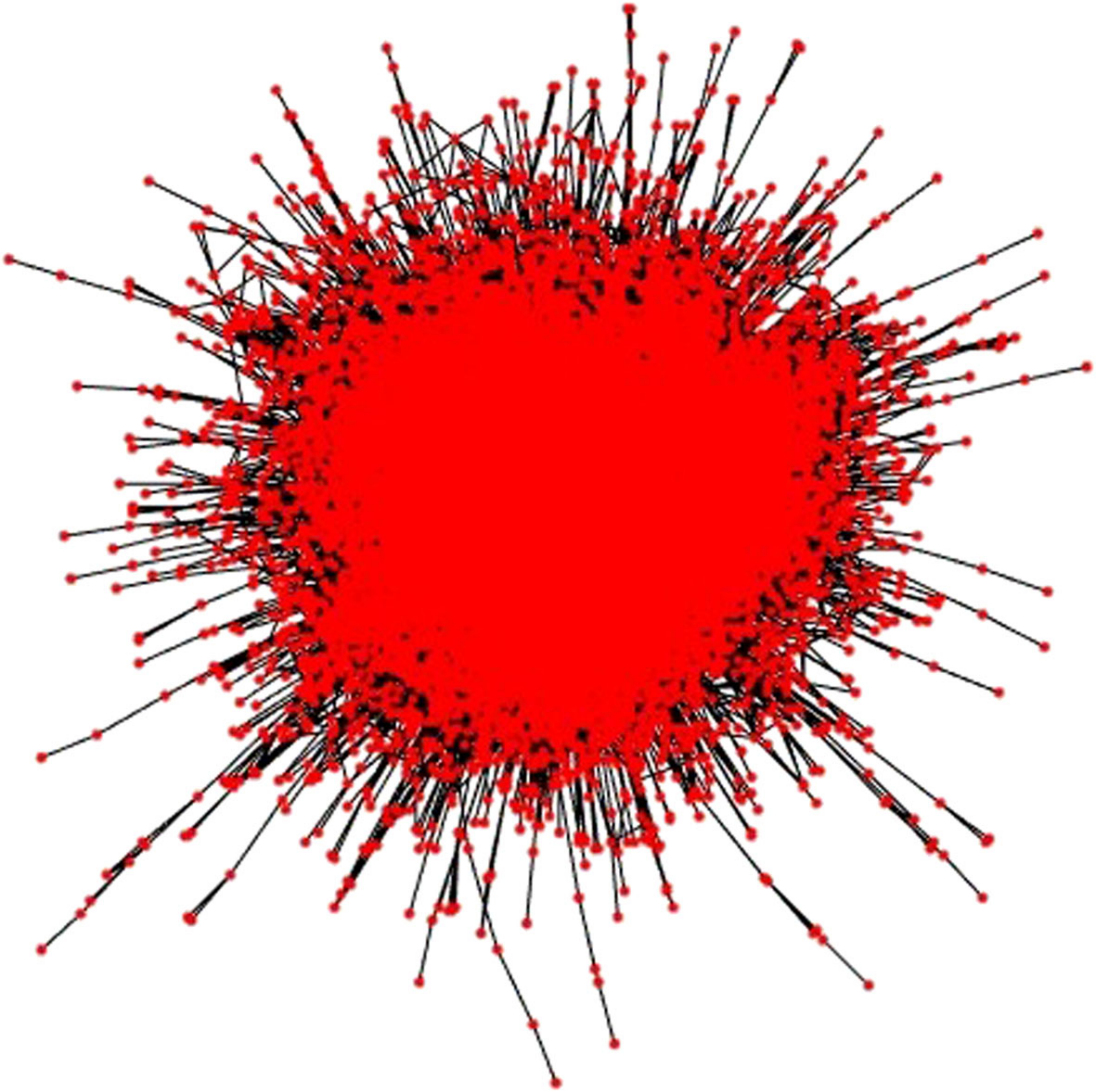
The arXiv HEP-Th (high energy physics theory) citation network. Its main connected component with 27770 nodes (articles) and 352807 arcs (citations)

**Fig. 4:**
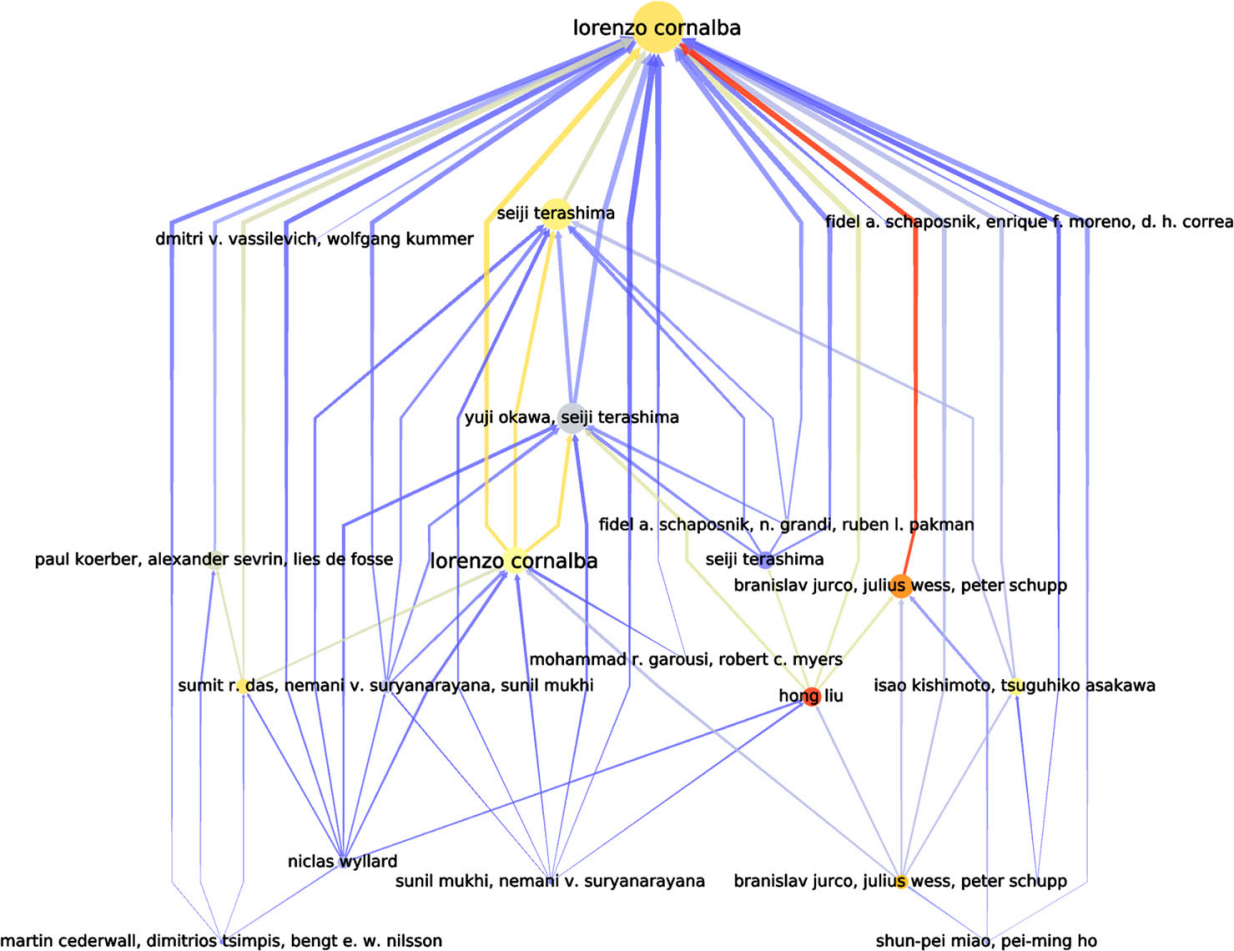
An example of the ascending flow metric in an excerpt of 22 nodes (60 edges) of our dataset, rooted by a publication by Lorenzo Cornalba. The size of nodes corresponds to their ascending flow in this subgraph. The color of nodes and edges (from *blue* to *red*) is actually their ascending flow in the real global dataset — we can see that Hong Liu’s publication has probably been a seed for more knowledge than of its ancestor Lorenzo Cornalba

The resulting graph (Fig. 3) is not acyclic due to the nature of publications in arXiv — some publications have been updated with cross-references to others. We can however consider this graph as pseudo-acyclic because the number and size of the cycles are limited (a few cycles of size 2 and 1 cycle of size 3). In our setting we simply remove those edges to keep the properties of a DAG. A resulting excerpt of the graph is shown in Fig. 4.

#### Correlations between measures on the whole archive

**Fig. 5:**
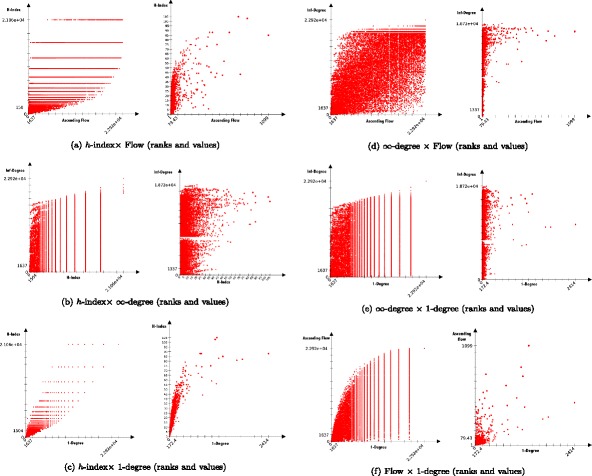
Comparative distribution of ranks and values among: ∙*h*-index (**a**, **b**, **c**), ∙ ascending flow (**a**, **d**, **f**), ∙ *∞*-degree – i.e. number total of generated publications (**b**, **d**, **e**), ∙ and 1-degree – i.e. number of citations of a publications (**c**, **e**, **f**). The plots illustrate the difference between what those statistics are measuring

As we have defined the generalized version of the number of citations in our framework and the *h*-index, we can compare these measures together using the Pearson (*ρ*) and Spearman (*r*_*s*_) correlation coefficients. We hold the following assumption: if the ascending flow can reconnect at least partially to the notion of degree and *h*-index, we can then validate the relevance of our framework. Results of the analysis are presented in Table 1 and Fig. 5.

**Table 1:** Comparison of Pearson coefficients (bottom left, correlation of values) and Spearman coefficient (top right, correlation of ranks) between all measures

*Pearson* *ρ* *Spearman* *r*_*s*_	*h*-index	ascending flow	*∞*-degree	1-degree	2-degree	5-degree	10-degree	20-degree	1-flow	2-flow	5-flow	10-flow	20-flow
*h*-index	-	0.821	0.765	0.958	0.954	0.849	0.770	0.765	0.776	0.809	0.807	0.807	0.807
Ascending flow	0.546	-	0.758	0.858	0.807	0.764	0.759	0.758	0.961	0.990	0.991	0.991	0.991
*∞*-degree	0.476	0.267	-	0.715	0.809	0.947	1.000	1.000	0.654	0.710	0.714	0.714	0.714
1-degree	0.768	0.648	0.265	-	0.920	0.794	0.719	0.715	0.856	0.863	0.860	0.860	0.860
2-degree	0.850	0.670	0.375	0.766	-	0.908	0.815	0.809	0.725	0.776	0.775	0.775	0.775
5-degree	0.626	0.347	0.856	0.367	0.546	-	0.952	0.947	0.657	0.714	0.716	0.716	0.716
10-degree	0.483	0.270	0.999	0.268	0.381	0.865	-	1.000	0.654	0.710	0.714	0.714	0.714
20-degree	0.476	0.267	1.000	0.265	0.375	0.856	0.999	-	0.654	0.710	0.714	0.714	0.714
1-flow	0.637	0.694	0.330	0.904	0.638	0.367	0.332	0.330	-	0.987	0.985	0.985	0.985
2-flow	0.664	0.814	0.337	0.892	0.712	0.390	0.339	0.337	0.969	-	1.000	1.000	1.000
5-flow	0.656	0.823	0.341	0.879	0.704	0.392	0.344	0.341	0.964	0.999	-	1.000	1.000
10-flow	0.656	0.823	0.341	0.879	0.704	0.392	0.344	0.341	0.964	0.999	1.000	-	1.000
20-flow	0.656	0.823	0.341	0.879	0.704	0.392	0.344	0.341	0.964	0.999	1.000	1.000	-

First, when comparing the *h*-index, the number of citations, and the total number of publications produced by a work, we can notice a clear difference on our four basic metrics — i.e. the number of citations (=1-degree), the number of publications generated (=*∞*-degree), the *h*-index and the ascending flow. We additionally varied the depth of degree and flow in {1,2,5,10,20,*∞*}. A second observation is that the limitation in depth of our measure is consistent with what we observe when limiting the depth of the *k*-degree (the most correlated *i*-flow for a *j*-degree is when *i*=*j*), and the higher *k* for the *k* degree, the more it diverges from the *k*-flow.

Our main observation, is, by value, the *h*-index is most correlated to the 2-degree. This makes complete sense, since the *h*-index is also limited in depth at 2 for which it considers a subset of publications. In contrast, when it comes to rankings, the *h*-index is most correlated to the 1-degree which is equivalent to the number of citations. Interestingly, our ascending flow also shares most correlation with the 2-degree as well and ranks with the 1-degree. This interesting effect may also be observed in Fig. 4 showing that most publications bringing influence to the source publication has done it already in depth two. The link between the *h*-index and the degree is further observable in Fig. 5.

In terms of computation, from *k*=2, the ranks obtained by the *k*-flow are *r*_*s*_=0.99 similar of those of the regular flow so when a gain of computation is needed, one can use *k*-diffuse version of the algorithm (Fig. 2).

#### Searching for a needle in the haystack

We observed all publications with *h*-index =6 (1054 publications in the dataset). With this sample of fixed *h*-index nodes, the ascending flow does not correlate well with other classical measures: *ρ*=0.136 with the 2-degree which was the most correlated value over the network with the ascending flow and the *h*-index, *ρ*=0.096 with the number of citations, *ρ*=0.228 with the total descending nodes (*∞*-degree).

**Fig. 6:**
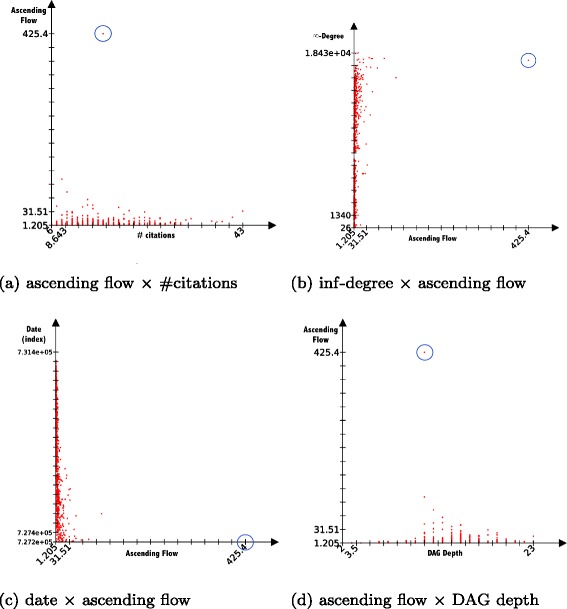
Comparative distributions of the ascending flow against: **a** 1-degree (i.e. number of citations **b** *∞*-degree (i.e. number total of generated publications), **c** date of publication, and **d** in blue the outlier detected by the ascending flow

However, if we plot the distribution of the flow coefficient (Fig. 6) we can notice one outlier node that cannot be noticed in any other distribution: this node happens to be the oldest publication (ID9201019^2^) among all publications with *h*-index =6. We also tested if the node was an outlier in all other measures, including date and DAG depth (which could be the longest if the node has deep descending nodes), but the work is not an outlier in any of those measures.

If we look in details at why this node received so much flow, we can “follow down” the path of maximum flow passed by its citing publications: we recursively take the edge that shares the maximum value until there is no other citing node. The path is only 22 edges long and the influence received by the nodes constantly increases up to 7 nodes before decreasing (Table 2 presents the statistics of the 8 first nodes of the path). Two articles in this path are the #2 and #3 most cited works in the whole archive!

**Table 2:** The chain of max flow citations from ID9201019: *The Coupling of Yang-Mills to Extended Objects*

Articles (index by date)	Flow	#citations	*h*-index
9201019:*The Coupling of Yang-Mills to Extended Objects*	425.446	16	6
9208055: *Putting String/Fivebrane Duality to the Test*	363.071	32	18
9304154: *Duality Symmetric Actions*	57.5917	229	44
9402032: *Dyon - Monopole Bound States*	84.5253	240	54
9408074: *A Strong Coupling Test of S-Duality*	32.3318	290	54
9510135: *Bound States Of Strings And p-Branes*	31.2979	775	88
9802109: *Gauge Theory Correlators from Non-Critical String Theory*	23.9841	1641	81
9802150: *Anti De Sitter Space And Holography*	14.0768,	1775	82
9803001: *Macroscopic strings as heavy quarks: Large-N gauge theory*	11.5337	.239	41

#### Comparisons of “similar” publications

**Fig. 7:**
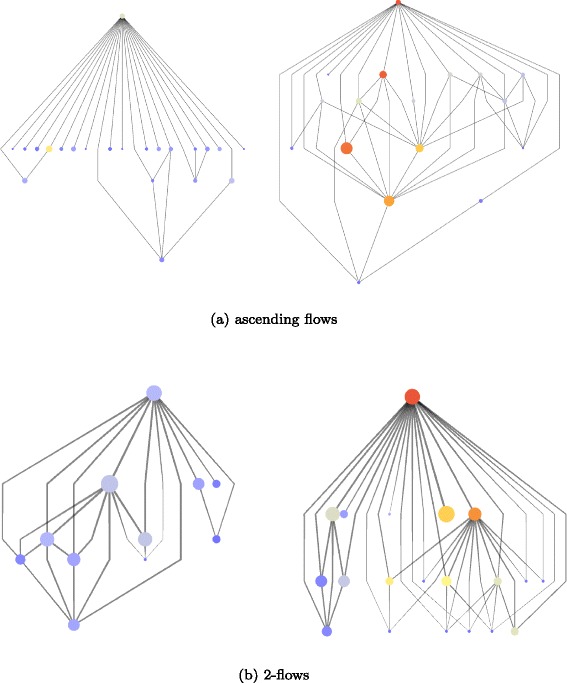
Comparison of direct citations of four publications with *h*-index=6. The *top node* is one original publication, and all other nodes its citing nodes. **a** Comparison of the general ascending flows with two extreme values: *left* ID9204026 (flow=425.4), *right* ID9201019 (flow=11.2). **b** Comparison of 2−flows with two extreme values: *left* ID9201079 (2−flow=2.3), *right* ID9201058 (2−flow=21.6). Relative node size (between couples of pictures) corresponds to *h*-index values for each node. Node color, from *blue* to *red*, corresponds to, **a** ascending flow, **b** 2-flow

Now we can compare publications of a same *h*-index and published around the same date which have very different flow measures. We took two publications with very different ascending flows: the first one shows a flow at 11.23 (Fig. 7a, left), while the second one displays a flow measure at 425.44 (Fig. 7a, right). Their in-degree does not vary that much (21 *vs.* 16 for the most influential), however the 2-degree makes the difference (151, vs. 707). That means in average, the publications citing the most influential work produce more than four times more citations in turn — average *h*-index is 3.2 vs. 10.6. Note also that our measure takes into account how the information is spread out, that can be captured by the total number of citations (i.e. arcs) going out from all nodes citing a publication (and this same publication). In the first case, we have 390 citation arcs, while we have 171 arcs in the second case.

We repeated the same experiment with two varying 2-flow measures (*h*-index =6 and similar date of publication): the first one is 2.25 with 10 citations (Fig. 7b, left), and the second one is 21.59 with 20 citation (Fig. 7b, right). The average *h*-index is actually higher in the least influential publication (3.45) than in the most influential publication (1.80). However, the most influential has seeded 102 publications (2-degree) for 107 arcs out, when the first one has seeded 68 publications (2-degree) for 182 arcs. The flow measures then capture much more details of the graph produced by citations than the *h*-index does.

## The citation network as a multiplex network

The ascending flow is based on the notion that knowledge diffuses equally among citations, with the implication that two works inspired by a third work borrow equally from it, and that one work citing two other works also borrows equally from each of them. However, there should be a notion of proximity between works that would translate *how* a work borrows from other works it is citing. Without parsing the whole text of a publication, there is no trivial and unbiased way to evaluate this notion of influence.

We have introduced in the previous section that each publication shows a different structure in the subgraph induced by its direct citing articles. We can consider all edges in this subgraph as one *layer of interaction* in the subgraph. Indeed, all edges in a direct citation subgraph share the common aspect that all nodes they connect directly cite the top-level node. If we consider our whole citation network as a unified collection of such subgraphs, we can consider that many layers of interactions induced by different publications overlap over the edges. Such a pattern can be captured through a *multiplex* formulation of our network (Kivelä et al. 2014). A multiplex network ${\mathcal {G}}$ can connect the same two nodes *p*,*q* over arcs *a*(*p*,*q*,*l*) in multiple layers *l* (see Fig. 12, left).

We present the citation network as a new application case for multiplex network analysis. It allows us to formulate a notion of multiplex flows that may be closer to the spirit of the flow of knowledge we want to capture. In addition, it also enables us to ask new types of questions in terms of relative influence of other work within a citation network, as we will illustrate in the following sections.

After introducing the definition of our multiplex network (“Preliminaries” section), we extend our ascending flow framework with three new multiplex versions: the aggregated flow, the sum flow, and the selective flow in “Multiplex extensions of the ascending flow” section, and implementation in “Dynamic implementation” section. We also study the edge entanglement (“Influence of layers in the multiplex citation network” section) in order to investigate influential works found from our experimental results in “Experimental results” section.

### Preliminaries

If we recall our original definition of the citation network (“Preliminaries” section), we consider a citation graph *G*=(*V*,*E*) in which a node *p*∈*V* represents a publication, and arcs (*p*,*q*)∈*E* when an article *p* cites an article *q*, that is considered a DAG.Each node *p* then bears a subgraph induced by all its citing articles. 

**Fig. 8:**
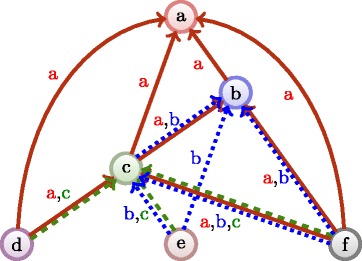
Illustration of the citation network from Fig. 1 transformed to a multiplex network, each citation creates its own layer of interaction

Figure 8 illustrates the concept introduced below.

#### **Definition 4**

A citation subgraph *G*_*p*_=(*V*_*p*_,*E*_*p*_) is induced by a node *p* such that $V_{p}={p \cup \mathcal {N}^- ({p})} \subset V$, and *E*_*p*_⊂*E* is such that, $\forall (q,r)\in V_{p}^{2},\;\; \exists a(q,r)\in E$.

Let consider, for each publication *p*, its induced citation subgraph *G*_*p*_=(*V*_*p*_,*E*_*p*_) of *G*, the multiplex citation network results in combining all individual subgraphs *G*_*p*_ together.

#### **Definition 5**

A multiplex network ${\cal {G}}=(V, {\mathcal {E}}, {\mathcal {L}})$ connects nodes (*p*,*q*) on different layers *l* such that arcs $a(p,q,l)\in \bigcup _{l \in {\mathcal {L}}}{\mathcal {E}}_{l}$. A multiplex citation network ${\cal {G}}=(V, {\mathcal {E}}, {\mathcal {L}})$ is defined such that ${\cal {G}} = \bigcup _{p}^{p \in V}{G_{p}}$, hence ${\mathcal {E}} = \bigcup _{p}^{p \in V}{E_{p}}$ and ${\mathcal {L}} = V$.

Note that an arc *a*(*p*,*q*,*l*) exists if and only if both *p* and *q* cite *l* or if *l*=*q*. As a consequence, the multiplex network once “flattened”^3^, has the exact same topology as the original citation network. The difference lies in the multiple edges.

#### **Definition 6**

A multiplex connection *α*(*p*,*q*) between nodes *p* and *q* is defined such that $\alpha (p,q) = \bigcup _{l}^{l \in {\mathcal {L}}_{p}}{a(p,q,l)},\;\; s.a.\;\; \exists a(p,q) \in {\mathcal {E}}$.

By extension of Definition 4, ${\cal {G}}_{p}=(V_{p}, {\mathcal {E}}_{p}, {\mathcal {L}}_{p})$ designates the multiplex subgraph induced by a node *p* and its citing articles $\mathcal {N}^- ({p})$. Note that this definition does not restrict layers to those corresponding to nodes of *V*_*p*_, since we are interested in observing co-citations.

#### **Definition 7**

A citation multiplex subgraph ${\cal {G}}_{p}=(V_{p}, {\mathcal {E}}_{p}, {\mathcal {L}}_{p})$ is induced by a node *p* with ${\mathcal {E}}_{p} \subset {\mathcal {E}}$ and ${\mathcal {L}}_{p} \subset {\mathcal {L}}$ such that, $\forall l \in {\mathcal {L}} \; \forall (q,r)\in V_{p}^{2},\;\; \exists a(q,r,l)\in {\mathcal {E}}$.

The co-citation relationship can also be characterized in the multiplex network. From the definition of our multiplex network, we can consider a node *p* to belong to different layers ${\mathcal {L}}(p)$, corresponding to the articles they cite, in addition to their own.

If we look at two articles such that *p* cites *q*, their multiplex connection *α*(*p*,*q*) corresponds to all the citations they have in common, hence the union of all arcs from *p* to *q* existing in all subgraphs $G_{l}, l \in {\mathcal {L}}(p,q) \subset V$: this corresponds to the co-citations of *p* and *q* in addition to *q* itself.

#### **Definition 8**

The set of layers ${\mathcal {L}}(p,q)$ connecting two nodes *p*,*q* is defined by ${\mathcal {L}}(p,q) = \{\mathcal {N}^+({p}) \cap \mathcal {N}^- ({q}), q\}$. By extension, we define ${\mathcal {L}}(p)=\{\bigcup _{q\in \mathcal {N}^- ({p})}{{\mathcal {L}}(p,q)}, p\}$.

Note that the number of layers of a multiplex connection $|{\mathcal {L}}(p,q)|$ (respectively, the number of layers to which a publication belongs to $|{\mathcal {L}}(p)|$) corresponds to the *multiplexity* of a connection (respectively, to the *multiplexity* of a node) (Podolny and Baron (1997)), i.e. the number of different layers connecting the pair of nodes *α*(*p*,*q*) (respectively connecting all pairs of node *α*(*p*,*q*),∀*q*∈*V*).

In our multiplex citation network, the notion of neighborhood remains the same as in the monoplex case (Definition 1). However we can refer to a different notion of multiplex degrees *δ* ^+^(*p*) and *δ* ^−^(*p*) that takes into account the number of arcs connecting a node to its neighborhood.

#### **Definition 9**

Denote the multiplex out-degree *δ* ^+^(*p*) (respectively the multiplex in-degree *δ* ^−^(*p*)) a node *p* in the multiplex network ${\mathcal {G}}$. $\delta ^{+}(p) = |a(p,q,l)|, \forall q \in V,\; \forall l \in {\mathcal {L}},\; s.a.\; \exists a(p,q,l) \in {\mathcal {E}}$ (respectively *δ* ^−^(*p*)=|*a*(*q*,*p*,*l*)|).

The degrees *d* ^+^(*p*) and *d* ^−^(*p*) still refer to the degree in the monoplex network *G*, i.e. the size of the neighborhood (Definition 1). We then introduce the degrees $d^{+}_{l}(p)$ and $d^{-}_{l}(p)$ corresponding to the degree in the subgraph *G*_*l*_, i.e. the number of arc adjacent to *p* on the layer *l*.

#### **Definition 10**

Denote the layer out-degree $\delta _{l}^{+}(p)$ (respectively the layer in-degree $\delta _{l}^{-}(p)$) a node *p* in the subgraph ${\mathcal {G}_{l}}$. $\delta _{l}^{+}(p) = |a(p,q,l)|, \forall q \in V,\; s.a.\; \exists a(p,q,l) \in {\mathcal {E}}$ (respectively $\delta _{l}^{-}(p) = |a(q,p,l)|$).

We will now turn to our advantage the fact that the definition of the multiplex subgraph induced by one publication *p*, as in Definition 7, implies that edges bear *all* the co-cited papers not restricted to the articles citing *p*. This allows the inspection of the subgraph directly without having to consider the whole graph, and the design of measures that can reduce the influence of an article in favor to other co-cited works.

### Multiplex extensions of the ascending flow

Since the multiplex formulation of flows in citation networks offers a wide range of possibilities for new measures of influence, we present three measures of flows: two straight-forward extensions of the ascending flow to the multiplex case, and one more elaborated.

#### Aggregated flow

This is a direct straight-forward extension of the ascending flow (“Ascending flow in citation networks” section). We keep the exact same definition but this time applied in a context with multiple edges.

6 $$ F(\mathcal{N}^- ({p}))=\sum\limits_{q \in \mathcal{N}^- ({p})} \frac{K(k_{q})}{\delta^{+}(k_{q})}+\lambda_{p}  $$

Driving this definition is a notion of proximity of sources between two publications: the more they share citations, the closer they can be considered, hence the citing publication is more influenced by the work it is citing. The cited publication will then receive a larger share of the flow gathered by the citing work in comparison to other works marginally cited. One advantage of this view is that, in a developing branch of knowledge, the most recent works sharing many citations with a publication will receive more flow — with the consequence of survey works dispatching more flow to recent works rather than fundamental works. Similar to “Ascending flow in citation networks” section we set *λ*_*p*_=1 the unit of contribution of one work.

**Fig. 9:**
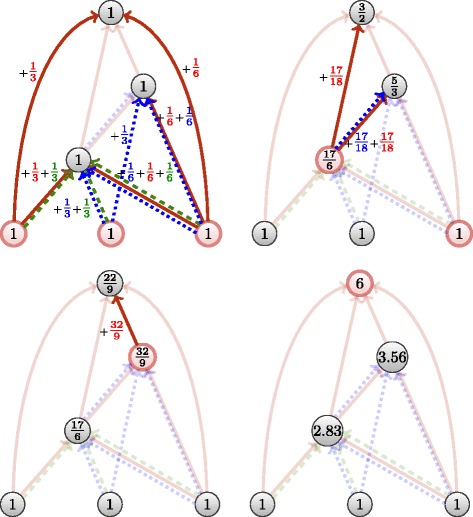
Aggregated flow in a multiplex network, step by step

Algorithm 2 shows the algorithm of the aggregated flow is exactly the same as the ascending flow, with the exception of an additional loop among layers (Algorithm 2 line 6). This loop correspond to the fact of going through all the layers of a multiple link between two citations. Computation is illustrated in Fig. 9. As discussed in “Ascending flow in citation networks” section, the complexity of Algorithm 2 is *Θ*(*m*) where *m* is the number of citations. Since the input is a multiplex network, in the worst case the number of links in the networks is equal to the number of nodes (layers) times the number of citations. Thus, the time-complexity of Algorithm 2 is *Θ*(*m* *n*).

#### Sum flow

This second extension of the ascending flow to a multiplex network consists in combining multiple monoplex versions of the ascending flow. Indeed, as we explain in “Preliminaries” section, for each publication *p*, we have an induced subgraph *G*_*p*_, for which we can compute an ascending flow. With this measure, influential nodes (such as surveys) send individually to each sources *only* the flow they directly received through their layer. It would correct the disadvantage of the aggregated flow by introducing some level of fairness. Its drawback is that such survey works will receive a boosted flow measure since they have greater chances to belong to many different layers.

Recall the definition of the ascending flow, adapted to a subgraph *G*_*l*_:

7 $$ F_{G_{l}}(\mathcal{N}^- ({p}))=\sum\limits_{q \in \mathcal{N}^- ({p})} \frac{K_{G_{l}}(k_{q})}{d^{+}_{G_{l}}(k_{q})}+\lambda_{(p,l)}  $$

The sum flow will simply sum ascending flows over all subgraphs composed of one layer, determined for a node *p* by:

8 $$ \begin{aligned} F_{{\cal{G}}}(p) & = \sum\limits_{l \in {\mathcal{L}}(p)} F_{G_{l}}(\mathcal{N}^- ({p})) \\ \lambda_{(p,l)} & = \frac{\lambda_{p}}{|{\mathcal{L}}(p)|} \end{aligned}  $$

If we set the parameter *λ*_(*p*,*l*)_=1, the contribution of one publication to the whole system will be exactly its number of citations, and a publication that cites lots of work will produce a lot of flow. In order to maintain constant the unit of contribution of a publication of a work, we set *λ*_*p*_=1 hence $\sum _{l}^{l \in {\mathcal {L}}(p)}\frac {1}{|{\mathcal {L}}(p)|} = 1$ such that a publication brings exactly 1 unit of contribution to the system.

**Fig. 10:**
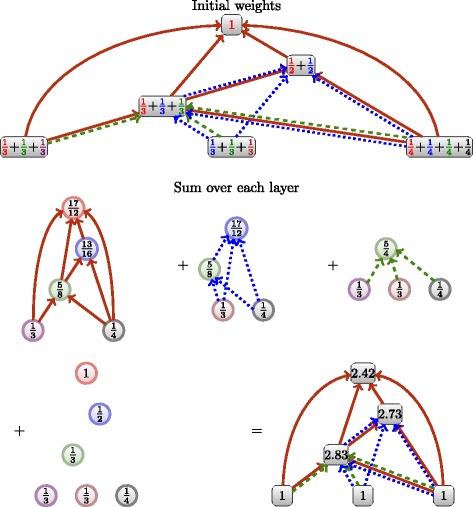
Illustration of the sum flow: we individually initialize weights to each node, depending on the number of layers each belongs to (including its own). We then compute the ascending flow for each layer (including individual), and sum the obtained weights

Algorithm 3 shows the algorithm of the sum flow is not a straightforward extension of the the ascending flow, as is Algorithm 2. The sum flow first constructs the citation subgraph for each layer, and compute the ascending flow on those layers afterwards to finally add them. Computation is illustrated in Fig. 10. As discussed in “Ascending flow in citation networks” section, the complexity of the Algorithm 1 is *Θ*(*m*) where *m* is the number of citations. As Algorithm 3 is iterated once per layer, thus per node, the complexity of our sum flow is *O*(*n* *m*). In practice, we can expect the size of the subgraphs to be much less than *m*.

#### Selective flow

This last extension attempts to correct both drawbacks of the previously defined flows. It is more restrictive and hybrids both of previous measures: one publication still brings one unit of contribution to the system. Not only flow circulates solely through one layer of interaction, but also the share generated by a layer will be transmitted along this layer.

Similarly to the ascending flow, we suppose that the flow first equally shares from one publication to all citations concerned. Then, within a pair of nodes, we suppose that all citations are equivalent.

9 $$ \begin{aligned} F_{{\cal{G}}_{l}}(\mathcal{N}^- ({p})) & = \lambda_{(p,l)} + \sum\limits_{q \in \mathcal{N}^- ({p})} \frac{K_{{\cal{G}}_{l}}(k_{q})}{d_{G_{l}}^{+}(k_{q})} \\ F_{{\cal{G}}}(p)& =\sum\limits_{l \in {\mathcal{L}}(p)} F_{{\cal{G}}_{l}}(\mathcal{N}^- ({p})) \\ \alpha_{(p, l)} & = \sum\limits_{j\in \mathcal{N}^+({p})} \frac{\lambda_{p}}{d^{+}(p)\times|{\mathcal{L}}(p,j)|} \end{aligned}  $$

Similarly to the sum flow, we set the unit of contribution of one publication to *λ*_*p*_=1, such that $\sum _{l \in {\mathcal {L}}(p)}\sum _{o\in \mathcal {N}^+({p})} \frac {\lambda _{p}}{d^{-}(p)\times |{\mathcal {L}}(p,o)|}=1$.

The less a cited work shares co-citation, the more it will be rewarded in flow. Contrarily to the aggregated flow, this is designed to increase the flow transmitted from works that brings originality from extra fields.

**Fig. 11:**
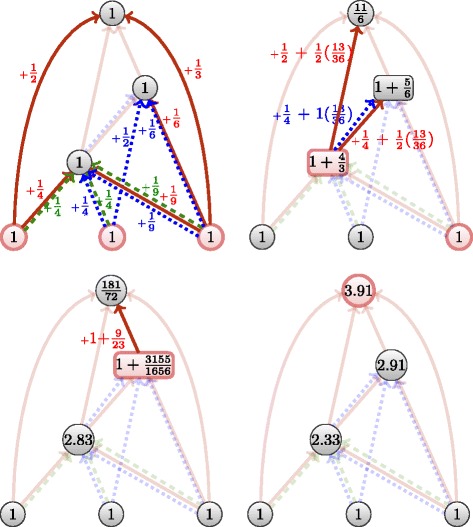
The selective flow, step by step. Notice that the weight a node received on one layer is only transmitted this layer only. Only a node’s initial unit is proportionally transmitted to all other layers

Principles are illustrated in Fig. 11 and the static algorithm is decribed in Algorithm 4. The algorithm behaves as a mix of the two previous multiplex flows in Algorithms 2 and 3. First, the selective flow will construct the different subgraphs of the original citation network (as in Algorithm 3). Then it will compute a kind of ascending flow on the whole multiplex graph (as in Algorithm 2). As such the complexity is also *O*(*m* *n*).

**Fig. 12:**
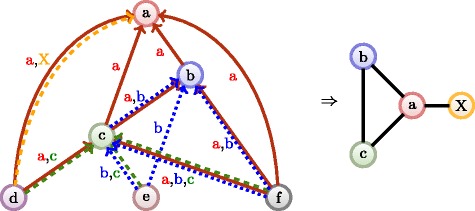
From multiplex citation network (*left*) to layer network (*right*). Note that we added a layer X in orange to illustrate that external citations can be included in the co-citation relationship. In the layer network, the layer *a* plays obviously the most central role as it is interacting with all other layers. Note that the layer network is undirected since the relationship becomes co-occurrence


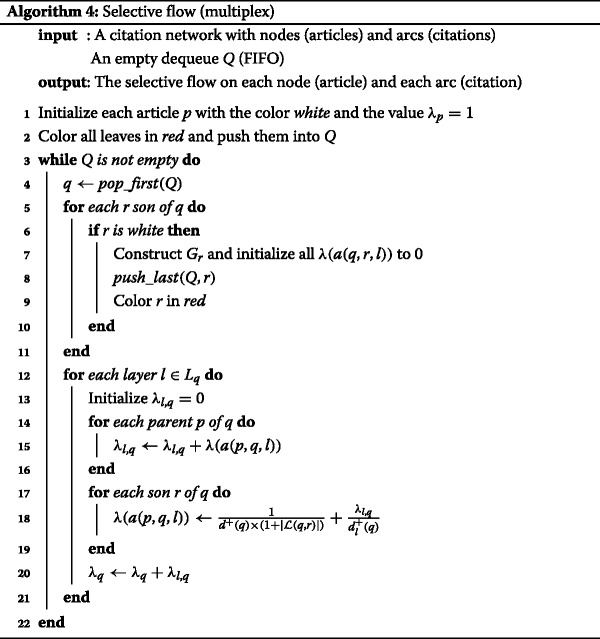


### Dynamic implementation

Although we presented static algorithms for computing our measure, actual dynamic implementation is much simpler. Similarly to the ascending flow, we consider the construction of our citation graph node by node in order to compute our measures. This means that we only need to compute a series of weights, and propagate them up when updating.

In the case of the ascending flow, we need to propagate in the whole hierarchy each time we add a node. For this reason, we proposed the *k*-diffuse property to ease the computation. The idea is exactly the same with the new measures, although additional details must be taken into account. Since we are using multiplex networks, two implementations are possible: either we take into account each edge colored by its layer individually, or we assign a set of colors (corresponding to layers) to each node and edge.

The aggregated flow propagates exactly the same way as the ascending flow, with the exception of the weighing scheme: there are just a little bit more edges to take into account. The newly added node *p* will propagate to *q* a weight $w_{agg}(p,q)=\frac {\lambda _{p}}{|{\mathcal {L}}(p,q)|}$ and same for *q* that will propagate with *λ*_*q*_=*w*_*agg*_(*p*,*q*).

For the sum flow, we first assign different weights for each color. The propagation then occurs similarly to the ascending flow. However, we need to compute an ascending propagation per each layer this newly added node belongs to (1 per citation). The newly added node *p* will propagate to *q* for each co-citation *l*∈(*p*,*q*) a weight $w_{sum}(p,q,l)=\frac {\lambda _{p}}{1+\delta ^{+}(q)}$, then *q* will propagate individually on each layer *l*, following the strategy of the ascending flow with *λ*_*q*,*l*_=*w*_*sum*_(*p*,*q*,*l*).

Finally, the selective flow is similar to the sum flow with a different weighting scheme. The newly added node *p* will propagate to *q* for each co-citation $l \in {\mathcal {L}}(p,q)$ a weight assigned per each edge $w_{sel}(p,q,l)=\frac {\lambda _{p}}{d^{+}(q)\times |{\mathcal {L}}(p,q)|}$, then *q* will propagate individually on each layer *l*, following the strategy of the ascending flow with *λ*_*q*,*l*_=*w*_*sel*_(*p*,*q*,*l*).

Although we do not propose a study of parameterization — with timely comparisons — here, all flow measures can be constrained similarly to the ascending flow, i.e. we can apply *evanescence* or *k*-diffusion to speed up implementation time.

### Influence of layers in the multiplex citation network

The multiplex definition of our citation network allows us many interesting studies, one of each is the analysis of edge entanglement (Renoust et al. 2014). Edge entanglement measures the overlapping of edges in a multiplex network. The entanglement index grows as multiple layers overlap together over multiple edges (similarly to hubs and authorities, but for layers).

One very interesting aspect of this approach is that it computes a second network, the network of overlapping layers (hereafter referred as the “layer network”, see Fig. 12, right), for which the entanglement measures may be computed. The visualization of this multiplex network gives interesting insights on how the layers are behaving together in the multiplex network. We use a publicly released online visualization tool^4^ (Detangler (Renoust et al. 2015)). It offers interaction in the multiplex network allowing to identify groups of particular nodes and their associated layers — as well as the other way around, particular groups of layers with their associated nodes (Renoust et al. 2015). Detangler also offers to study many other aspects of the multiplex network by changing metrics, taking weights into account, and measuring global cohesion of groups of publications (Renoust et al. 2014). For the sake of simplicity, we will only look at the exploration of the layers in association with citing articles as it already brings insights.

This is particularly useful to investigate a multiplex subgraph ${\mathcal {G}}_{p}$ induced by a publication *p*. For example, the topology of the layer network can indicate subgroups of citations that tend to be co-cited together. With interaction, we can as well identify a subgroup of publications that tend to co-cite the same subgroup of citations. Since ${\mathcal {G}}_{p}$ is by definition induced by *p*, we also expect the publication *p* to be the most overlapping publication. However, the co-citing relationships including many additional layers, we can identify other fundamental works that are co-cited in the network.

### Experimental results

We follow up on our experiment in “Experimental results” section, and computed values of all new multiplex flows on the arXiv HEP-Th dataset.

#### Correlations between measures on the whole archive

**Fig. 13:**
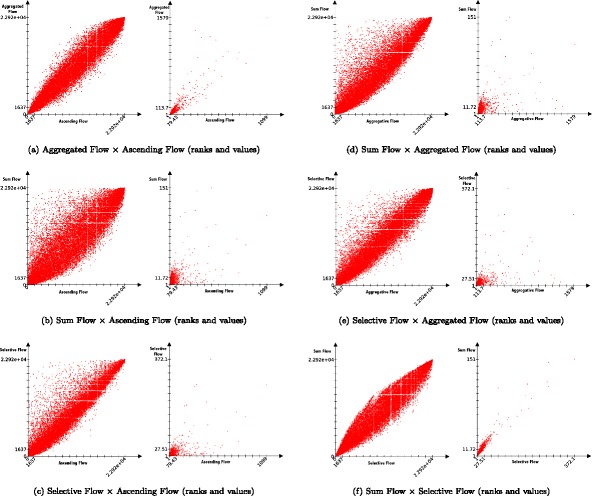
Comparative distribution of ranks and values among: ∙ ascending flow (**a**, **b**, **c**), ∙ aggregated flow (**a**, **d**, **e**), ∙ sum flow (**b**, **d**, **f**), ∙ and selective flow (**c**, **e**, **f**). The plots illustrate the difference between what those statistics are measuring

We compare the Pearson *ρ* and Spearman *r*_*s*_ correlation coefficients of these measures, as well with our original ascending flow. Results of the analysis are presented in Table 3 and Fig. 13. The first thing we can notice is that the multiplexity is not correlated to any other measure. By construction of our network, the multiplexity would be highly correlated with the out-degree of a publication (the number of articles cited), since a publication cannot connect through more layers than it is citing. However it has no clear link with its in-degree (its own number of citations).

**Table 3:** Comparison of Pearson coefficients (bottom left, correlation of values) and Spearman coefficient (top right, correlation of ranks) between all measures

*Spearman* *r*_*s*_	Monoplex			Multiplex				
*Pearson* *ρ*	*h*-index	1-degree	inf-degree	Ascending flow	Aggregated flow	Sum flow	Selective flow	Multiplexity
*h*-index	-	0.958	0.765	0.821	0.846	0.873	0.836	0.330
1Degree	0.768	-	0.715	0.858	0.880	0.939	0.899	0.322
InfDegreeCoeff	0.476	0.265	-	0.758	0.748	0.661	0.680	0.038
AscendingFlow	0.546	0.648	0.267	-	0.975	0.918	0.967	-0.011
AggregatedFlow	0.541	0.586	0.248	0.912	-	0.952	0.962	0.110
SumFlow	0.786	0.953	0.322	0.628	0.611	-	0.972	0.226
SelectiveFlow	0.665	0.957	0.257	0.682	0.624	0.946	-	0.072
Multiplexity	0.248	0.168	-0.044	-0.005	0.030	0.191	0.070	-

The second remark is that the original ascending flow and the aggregated flow are very correlated (*ρ*=0.912). This is of course expected since the definition of the aggregated flow directly extends the ascending flow. Both are actually equivalent when a publication shares exactly the same number of co-citations with all its citing articles. A look at Fig. 13 (a) shows that many cases differ in practice.

**Fig. 14:**
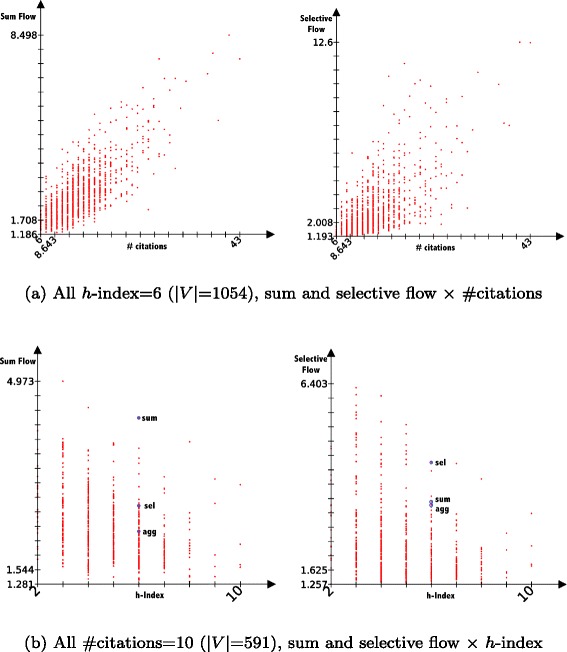
Comparison of sum and selective flows among **a** all publication with *h*-index=6 **b** all publication with #citations = 10. *Circled in blue* are the publications with the maximum aggregated, selective and sum flows of the subset, corresponding to the graphs shown in figure

Similarly, we can explain the correlation between the selective and sum flows (*ρ*=0.946), the selective flow inheriting from the sum flow. What came as a surprise is that both measures actually highly correlate with the number of citations (*ρ*>0.95 in both cases). However, our correlation coefficients are not robust (Devlin et al. 1975) and the heavy tailed distribution of our measures render their interpretation difficult (Devlin et al. 1975) (plots in Fig. 13 show that most of the 29+K values are usually tight in a dense group around low values). Nevertheless, if we look at correlations within the subset of *h*-index =6 publications, the values are widely spread (see Fig. 14a).

#### Searching for multiple needles in the haystack

To illustrate the difference between the multiplex flows, we can explore a subset of publications with a fixed number of citations (arbitrarily set to 10, corresponding to 591 publications). We can observe in the distributions (Fig. 14b) between our sum and selective flows and the *h*-index that values widely vary.

**Fig. 15:**
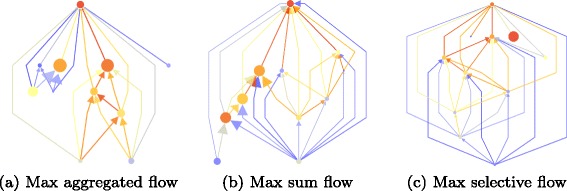
The induced subgraphs of the top publication identified by each of the three multiplex flows, in the subset (#citations=10, *h*-index=6). The size of nodes correspond to their number of citations, the size of edges correspond to their number of co-citations. The color of nodes and edges correspond to the flow measures transiting through each node/edge (**a**: aggregated, **b**: sum, **c**: selective), minimum is *blue*, maximum is *red*. In the case of the sum and selective flow, we can notice clear differences between node size and color mapping emphasizing the differences between the flow measures and the #citations

To continue our investigation, we can take a look at all publications in this subset having *h*-index =6. The positions of the nodes with maximum flows show very different cases (identified from the blue circles in Fig. 14b). We put the three works for illustration in Fig. 15 with their information in Table 4.

**Table 4:** Information of the most relevant publication and their main influence as discovered with the different multiplex flows among the (#citations=10, *h*-index=6) publication subset

	*Top node*	*Most flow received from*
*Aggregated Flow*	ID9503097: *Inflation With Variable Omega*	ID9802030:*Open Inflation Without False Vacua*
*Sum Flow*	ID9303050: *Conformal Turbulance with Boundary*	ID9307091: *Solutions of Conformal Turbulence on a Half Plane*
*Selective Flow*	ID9503097: *Infrared regularization of non-Abelian gauge theories*	ID:0728589*On the renormalizability and unitarity of the Curci-Ferrari model*

#### Exploration of individual induced subgraphs

We have identified three interesting publications, and can now investigate their induced subgraphs. We will pay a special attention to how other publications are cited in their subgraphs. The idea is to find whether there are other very influent works that inspired this subset of publications or not. We will also check if we can find sub-communities of co-citations. Layers (hence cited articles) that have a relatively high entanglement index may either form subgroups of layers or be some influent work. Entanglement indices are computed normalized over the whole multiplex network. Relatively high entanglement indices are groups of connected layers (in the layer-interaction network) that display higher values than their surrounding.

**Fig. 16:**
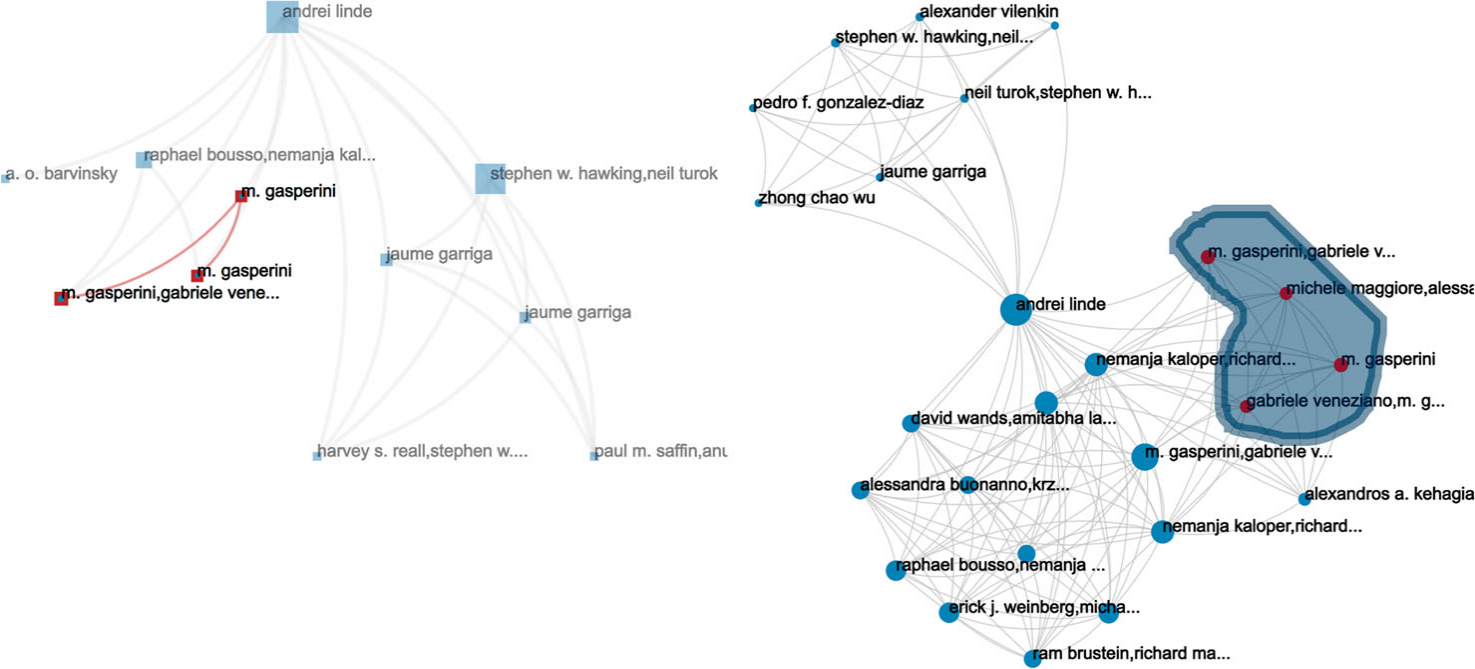
Detangler (Renoust et al. 2015) view of the subgraph induced by ID9503097 (aggregated flow). On the *left* the citation network (size is the aggregated flow), on the *right* the layer network (size is the entanglement index). Nodes are labeled with their authors’ name. We can notice that the layer network splits into two parts, the rightmost part forming another subnetwork. A subgroup of nodes consistently shows one same author. Selection from highlights the citing article. Two articles citing a third one: all include the the same author

**Fig. 17:**
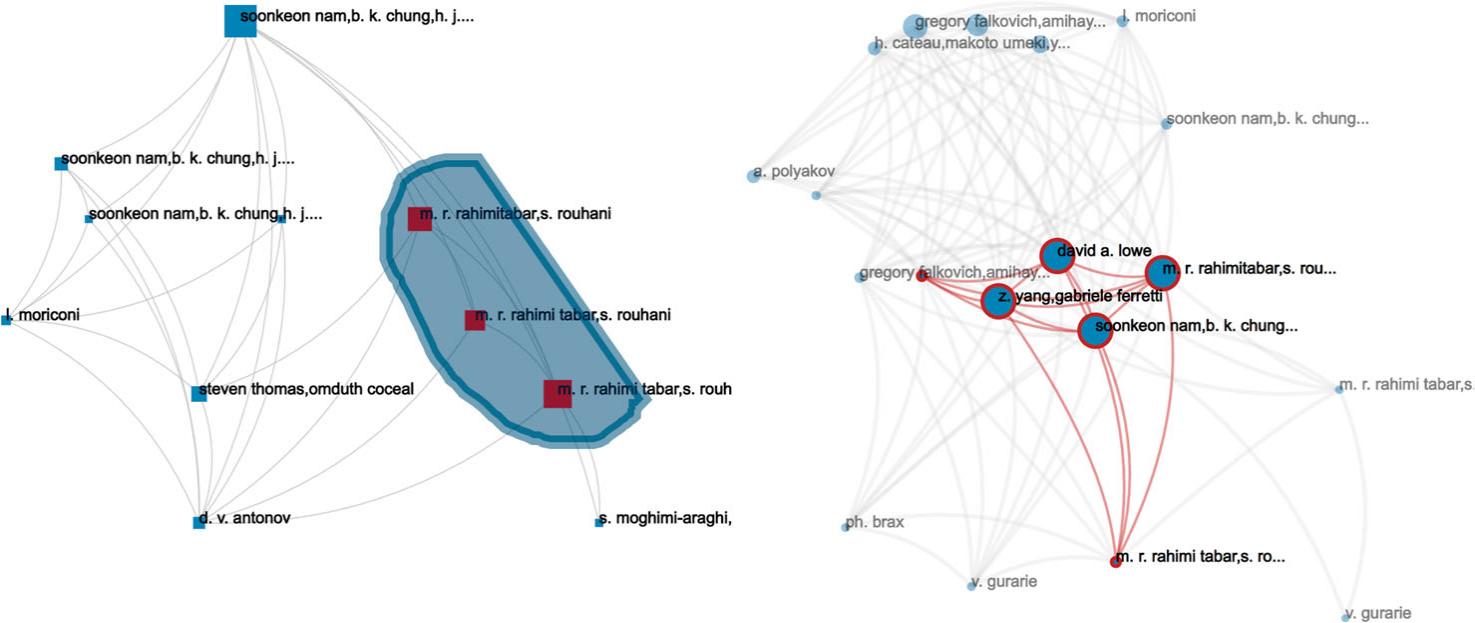
Detangler (Renoust et al. 2015) view of the subgraph induced by ID9303050 (sum flow). On the *left* the citation network (size is the sum flow), on the *right* the layer network (size is the entanglement index). Nodes are labeled with their authors’ name. The topology of the layer network suggests two intertwined communities (*top* and *bottom*). In the citation network (*left*) we select the three citing articles authored by the same group (with high sum flow). The corresponding co-cited articles (shown on the *right*) display four main cited articles (and two marginal ones). Only one of the main is a self-citation — and one marginal as well

**Fig. 18:**
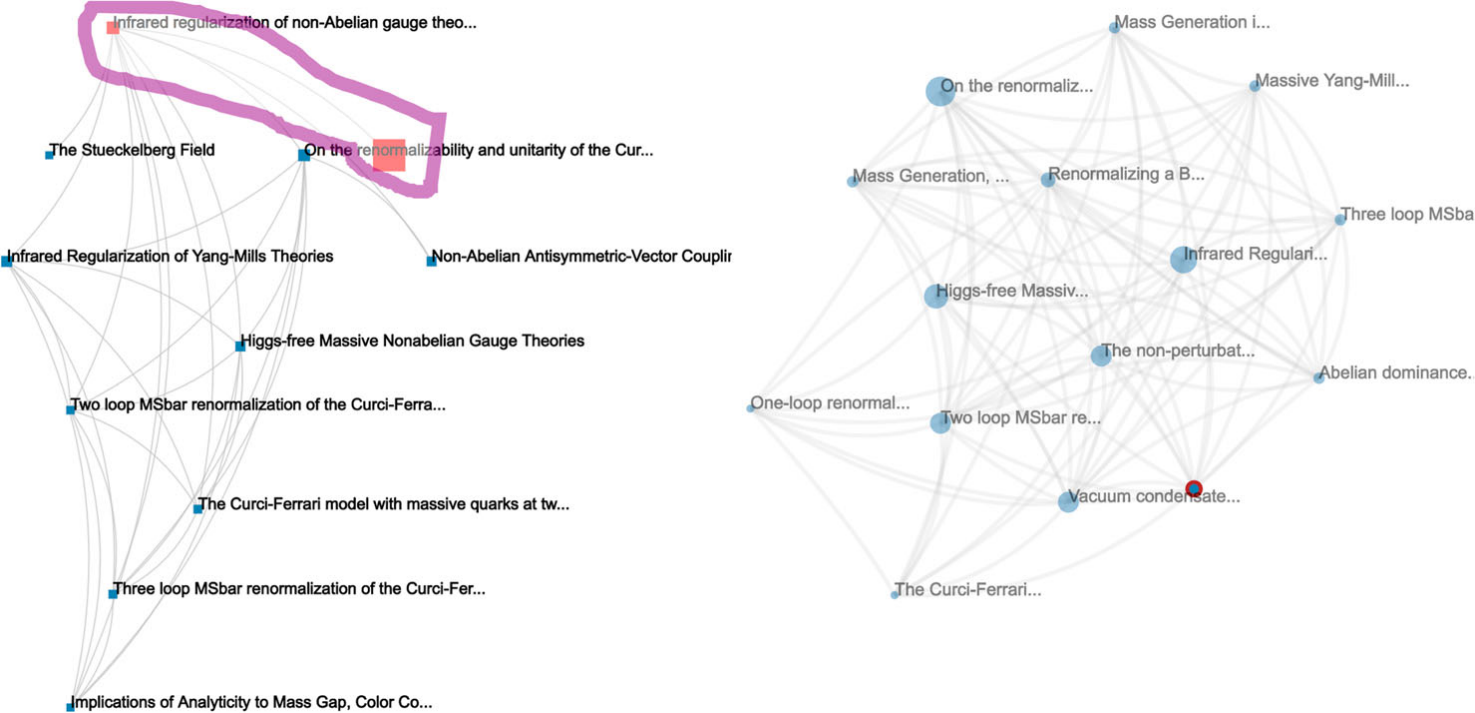
Detangler (Renoust et al. 2015) view of the subgraph induced by ID9509084 (selective flow). On the *left* the citation network (size is the selective flow), on the *right* the layer network (size is the entanglement index). Nodes are labeled with article titles. One citing article displays a very high selective flow, however the original publication (*on top*) bears a much lower value. We select both to highlight their co-citations and only the original publication pops out: the influence of this work must come from many other publications

In the following networks (Figs. 16, 17 and 18), we will present on the left the publication network of citing articles and on the right the layer network of cited articles. The publication networks are laid out with Sugiyama et al.’s algorithm for DAGs (Sugiyama et al. 1981), and the layer network with a force layout (since the relationship is co-occurrence). The size of the publication nodes corresponds to the metric that identified the publication, and the size of the layer nodes corresponds to their entanglement index.

If we look at the subgraph identified by the aggregated flow (ID9503097), we can clearly see two communities of cited articles in the layer network (Fig. 16, right) corresponding to the two separated branches of citing articles (Fig. 16, left). One community of layer nodes is also almost separated in two. One subgroup of layer nodes correspond to a smaller group of three publications, two citing another third. A small investigation on this group of publications actually shows that they all have one author in common (M. Gasperini), and that a fair amount of the corresponding subgroup of cited articles have been also published by this author: this may be a case of excessive self-citations.

Now we can look at the subgraph identified by the sum flow (ID9303050). The topology of the layer network suggests two intertwined communities. A fair amount of external nodes shows relatively high entanglement index. They are cited by almost all the other nodes (from selection of those nodes). From the publication network, three other publications show relatively high value of sum flow. A little inspection shows us that those three are from the same authors (M. Rahimitabar and S. Rouhani). From selection, we can see they co-cite mainly four publications (bigger highlighted nodes on the right in Fig. 17), but this time among these there is only one self-citation!

Finally, we can investigate the subgraph identified by the selective flow. The layer network shows a very different topology: it is almost one big connected clique. All the major layer nodes correspond to citing publications nodes. Additional layer nodes almost always connect only one pair of publications each. Manual inspection does not show dominant authors or much self citation in this group, it is actually very well balanced. One citing publication node pops out as it displays a very high flow value. We could suspect that this node is the reason this subgraph was detected in the first place. However, we know from Fig. 18 that most of the top node’s flow does not come from this citing article. Indeed, if we select this node and its citing node, only the citing node’s layer is displayed: the top publication will only receive from this node the share of citation that corresponds to its layer. The citing article must have received influence from many other works.

We inspected the network, and it appears overall flawless: no major work is often co-cited with the top publication; no other citing article brings an imbalanced larger share of flow to the publication; no dominant authors and no excessive self-citation.

## Discussion and conclusion

### On the notion of flow in citation networks

We have shown that the production and diffusion of knowledge can be modeled in a recursive framework that studies flows in DAGs, with a natural interpretation of the notion stream of knowledge. The framework allows for other known metrics to be embedded, and for efficient computation on large dynamic graphs. By comparing the ascendant flow with the *h*-index we clearly see some level of correlation, in regards to the meaningfulness of the Pearson coefficient. Our measure’s interpretation is straightforward, and this correlation goes in favor of the relevance of the *h*-index, and in some extent also to the number of citations. But we do not fully correlate with the *h*-index, and many cases that are oversimplified by the *h*-index can be more accurately described by the ascending flow. In addition, the flow can be used to find paths in the citation network, trying to explain the sources of influence. One interesting extension of this study would be to characterize if a node genuinely receives flow from being cited by a large crowd, or maybe once it has spread across different (sub)domains of science, or from being cited by very influent works.

We found cases with large differences among ascending flow for publications with the same *h*-index. The *h*-index gives a rough estimation of a publication’s production of knowledge, but it does not take into account how each citation refers to the original work. The ascending flow measure, even 2−diffuse, is reinforced by two factors. A first one is something similar to a “community” effect in citations, i.e. when the citations produced also cite each other in relative proportion, in comparison to citations “outside” that “community” of citations. For example, this happens when a paper has an influence in developing a community of research: the larger the community, the greater the flow. The second effect gets more relevant as the depth of diffusion is greater. It is somewhat close to the hubs and authorities effect: the more citations a paper gets from influential papers the more influential it will become.

The interpretation of flow we propose is much more flexible than the *h*-index, and can fairly support a wide range of parameters to conduct further experiments (such as additional weights, edge filtering, depth of influence, etc.). More than a metric, when studying the influence of a work (or a collection of works), we argue that the structure of the flow of knowledge it produces, i.e. the DAG generated by a publication and its citations should be taken into account.

### On the multiplex interpretation of the citation network

We also proposed to extend this model to a multiplex view of the citation network such as the edges bear the additional information of co-citations. The ascending flow fits well in this new model, and we could define three original flows. Although those interpretations of the flow are not independent of previous metrics, use cases show that it brings finer details, all things being equal. One important direction of future works would be a better study of the design space offered for other measures. We have chosen three interpretations of multiplex flow for our application case, but we will need to study their limits, dynamic/static implementation characteristics, and properties so we can better control and implement them. Implementation of the multiplex measure is a naive extension of the monoplex version, but we surely have room for optimization.

This multiplex view of the network would support many extensions to better fit the idea of knowledge diffusion, and from publication analysis, we could find the number of references to each citation within an article and use it to weight our individual interaction. Text analysis is also another direction that could bring topical proximity to even better refine the networks (as well demonstrated by Dong et al. (2012)).

Interacting with the publication subgraphs on Detangler brought really interesting insights from the content of an article’s co-citation network. This could be a helpful tool to suggest important new readings when evaluating a publication from a specific domain, or even to unearth very influential works on a specific venue. The questions we asked ourselves on self-citations discovered through interaction also opened us to new possibilities: we could use individual authors as a way to define a layer of interactions (such as in the work Boden et al. (2012)), and consider in the same time a co-cited author network (somewhat close to the spirit of one of Detangler’s use-case (Renoust et al. 2015)), forming a multiple-multiplex network. Our introduction of the multiplex DAG also invites us to study with them all known multiplex measure and extend other DAG measures to the multiplex case.

### Conclusion

We have introduced the notion of flow in citation networks, proposed a measure the ascending flow, discussed its implementation and experimented with it in the arXiv HEP-Th dataset. This notion of flow allowed us to propose the new approach of the citation network as a multiplex DAG for which we offered three measures — the aggregated, sum and selective flows — with implementation and experiments on the same dataset. We also leveraged visualization to explore our results and discovered interesting insights.

Although our study does not hold for an evaluation for which a comparison with many other metrics and regression will be necessary, we still have set and validated the basis of our framework. Not being high-energy theoretical physicists ourselves, it remains somewhat difficult to directly interpret the results, nonetheless exploration of our results showed interesting insights.

It will be important in the future to carefully curate controlled datasets for which we can expect results and apply former validation. As such, it would be interesting to examine the insights we could extract from a citation network inherited from all publications of a conference venue; to explore neighborhoods of important works such as surveys; to suggest new relevant works from a set of publications; or to analyze the genesis of two merging domains.

We wish to extend our study to other databases, such as DBLP or web of science, and bring the analysis to the authors themselves — although ambiguity in author names is quite challenging. It will be interesting to observe the influence of Nobel prizes for example, find their most influent work and their own influence.

Finally, this framework is not limited to citation networks, but can apply to many other type of transferred information: among our future works is the application to the analysis of news documents. Indeed, DAGs also apply to the study of closely related documents — even if there is no citation relationship, the time dependency between closely related documents can maintain the DAG assumption. It could be an interesting way to find the sources of information, and characterize the impact of news events.

## Endnotes

^1^ Available at: http://snap.stanford.edu/data/cit-HepTh.html.

^2^ *The Coupling of Yang-Mills to Extended Objects* (Dixon et al. 1992), by Dixon, Duff and Sezgin published on arXiv in 1992. And it has well deserved another citation!

^3^ By “flattening”, we mean the process of projecting the multiplex network such that one edge only is shared between two nodes in order to obtain a monoplex version of the multiplex network.

^4^ https://github.com/renoust/Detangler/tree/demo.
