# Erratum to: Multiplex flows in citation networks

**DOI:** 10.1007/s41109-017-0045-0

**Published:** 2017-08-29

**Authors:** Benjamin Renoust, Vivek Claver, Jean-François Baffier

**Affiliations:** 1 National Institute of Informatics, Tokyo, Japan; 2 JST-ERATO Kawarabayashi Large Graph project, Tokyo, Japan; 3 Japanese-French Laboratory for Informatics CNRS UMI 3527, Tokyo, Japan; 4 University of California Berkeley, Berkeley, USA

## Erratum

The original article (Renoust et al., 2017) contains an error carried forward by the Production team handling this article whereby Fig. 2, Fig. 3 and Fig. 4 were mistakenly placed in the incorrect order and with the incorrect captions.

The original article has now been updated to correct the order of these figures and place them with their respective correct captions.
